# Treatment outcomes of esophageal cancer in Eastern Africa: protocol of a multi-center, prospective, observational, open cohort study

**DOI:** 10.1186/s12885-021-09124-5

**Published:** 2022-01-19

**Authors:** Geoffrey C. Buckle, Alita Mrema, Michael Mwachiro, Yona Ringo, Msiba Selekwa, Gift Mulima, Fatma F. Some, Blandina T. Mmbaga, Gita N. Mody, Li Zhang, Alan Paciorek, Larry Akoko, Paul Ayuo, Stephen Burgert, Elizabeth Bukusi, Anthony Charles, Winnie Chepkemoi, Gladys Chesumbai, Bongani Kaimila, Aida Kenseko, Kitembo Salum Kibwana, David Koech, Caren Macharia, Ezekiel N. Moirana, Beatrice Paul Mushi, Alex Mremi, Julius Mwaiselage, Ally Mwanga, Jerry Ndumbalo, Gissela Nvakunga, Mamsau Ngoma, Margaret Oduor, Mark Oloo, Jesse Opakas, Robert Parker, Saruni Seno, Ande Salima, Furaha Servent, Andrew Wandera, Kate D. Westmoreland, Russell E. White, Brittney Williams, Elia J. Mmbaga, Katherine Van Loon, of the African Esophageal Cancer Consortium (AfrECC)

**Affiliations:** 1 UCSF Helen Diller Family Comprehensive Cancer Center, University of California, San Francisco, 505 Parnassus Ave, M1296, San Francsico, CA 94143 USA; 2 Ocean Road Cancer Institute, Dar es Salaam, Tanzania; 3 Tenwek Hospital, Bomet, Kenya; 4 Muhimbili National Hospital, Dar es Salaam, Tanzania; 5 Muhimbili University of Health and Allied Sciences, Dar es Salaam, Tanzania; 6 Kamuzu Central Hospital, Lilongwe, Malawi; 7 Moi University School of Medicine, Eldoret, Kenya; 8 Kilimanjaro Clinical Research Institute, Kilimanjaro Christian Medical Centre, Moshi, Tanzania; 9 Kilimanjaro Christian Medical University College, Moshi, Tanzania; 10 University of North Carolina, Chapel Hill, USA; 11 Kenya Medical Research Institute, Nairobi, Kenya; 12 UNC-Project Malawi, Lilongwe, Malawi; 13 Moi Teaching and Referral Hospital, Eldoret, Kenya; 14 Kilimanjaro Christian Medical Centre, Moshi, Tanzania; 15 Warren Alpert School of Medicine at Brown University, Providence, RI USA

**Keywords:** Esophageal squamous cell carcinoma, Esophageal cancer, Survival, Quality of life, Comparative effectiveness, Africa, Eastern Africa

## Abstract

**Background:**

Esophageal squamous cell carcinoma (ESCC) is a major cause of cancer morbidity and mortality in Eastern Africa. The majority of patients with ESCC in Eastern Africa present with advanced disease at the time of diagnosis. Several palliative interventions for ESCC are currently in use within the region, including chemotherapy, radiation therapy with and without chemotherapy, and esophageal stenting with self-expandable metallic stents; however, the comparative effectiveness of these interventions in a low resource setting has yet to be examined.

**Methods:**

This prospective, observational, multi-center, open cohort study aims to describe the therapeutic landscape of ESCC in Eastern Africa and investigate the outcomes of different treatment strategies within the region. The 4.5-year study will recruit at a total of six sites in Kenya, Malawi and Tanzania (Ocean Road Cancer Institute and Muhimbili National Hospital in Dar es Salaam, Tanzania; Kilimanjaro Christian Medical Center in Moshi, Tanzania; Tenwek Hospital in Bomet, Kenya; Moi Teaching and Referral Hospital in Eldoret, Kenya; and Kamuzu Central Hospital in Lilongwe, Malawi). Treatment outcomes that will be evaluated include overall survival, quality of life (QOL) and safety. All patients (≥18 years old) who present to participating sites with a histopathologically-confirmed or presumptive clinical diagnosis of ESCC based on endoscopy or barium swallow will be recruited to participate. Key clinical and treatment-related data including standardized QOL metrics will be collected at study enrollment, 1 month following treatment, 3 months following treatment, and thereafter at 3-month intervals until death. Vital status and QOL data will be collected through mobile phone outreach.

**Discussion:**

This study will be the first study to prospectively compare ESCC treatment strategies in Eastern Africa, and the first to investigate QOL benefits associated with different treatments in sub-Saharan Africa. Findings from this study will help define optimal management strategies for ESCC in Eastern Africa and other resource-limited settings and will serve as a benchmark for future research.

**Trial registration:**

This study was retrospectively registered with the ClinicalTrials.gov database on December 15, 2021, NCT05177393.

**Supplementary Information:**

The online version contains supplementary material available at 10.1186/s12885-021-09124-5.

## Background

Esophageal cancer (EC) is one of the leading causes of cancer morbidity and mortality worldwide. Most recent estimates from GLOBOCAN report that EC is the eight most common cancer globally with 604,000 annual incident cases and the sixth leading cause of cancer-related mortality with 544,000 deaths each year [1]. Nearly 80% of all EC cases occur in low- and middle-income countries (LMICs), where esophageal squamous cell carcinoma (ESCC) is the dominant histology [2]. Incidence rates for ESCC exhibit substantial geographic variation globally with several regions impacted by a disproportionately high burden of this disease. Eastern Africa has been identified as one of these geographic “hot spots,” along with north-eastern Iran, Central Asia, north-central China, and southern South America [2, 3]. In Eastern Africa, EC is the fifth leading cause of cancer mortality [1], with ESCC comprising 90% of cases [4].

Limited research has been published on the treatment of ESCC in sub-Saharan Africa [5]. In this setting, more than 90% of patients present with advanced or inoperable disease, and few are candidates for treatment with curative intent [6–8]. Palliative treatment strategies for advanced ESCC thus dominate the therapeutic landscape and include stenting for malignant obstruction with self-expandable metallic stents (SEMS), radiotherapy, chemo-radiotherapy, and brachytherapy. Use of each of these treatment modalities is supported by data that originates from high-income countries (HICs) [9]. The unique challenges of delivering care in resource-constrained settings, however, limit the generalizability of these findings to many African settings. In a previous systematic review of all studies on EC treatment in Africa [5], we identified only four prospective case series evaluating treatment of advanced ESCC in Eastern Africa, three of which reported outcomes of palliative stenting at two sites in Kenya and Malawi [10–12] and one that reported outcomes of palliative radiation in Ethiopa [13]. To date, no study has prospectively compared treatment modalities for advanced ESCC in Eastern Africa. Furthermore, little is known regarding the impact of different ESCC treatments on patients’ quality of life (QOL) in this setting. Of all the studies identified in our prior systematic review, only one examined QOL as an outcome metric [13].

With recent enhancement of cancer services in sub-Saharan Africa, the absence of context-specific data on treatment outcomes for ESCC is manifest. Expanded availability of chemotherapy, radiotherapy, and SEMS in Eastern Africa [14], has brought to light important questions regarding optimal management strategies for ESCC in this context. To address these questions, we developed a study protocol that aims to investigate the therapeutic landscape of ESCC in Eastern Africa and compare outcomes of different treatment approaches in use across the region. The Treatment Outcomes of Esophageal Cancer in Eastern Africa (TOEC-Eastern Africa) study is a prospective, observational, open cohort study of treatment strategies for ESCC that will recruit patients at six centers in Kenya, Malawi and Tanzania. This multi-center study has been established as a collaborative effort within the African Esophageal Cancer Consortium (AfrECC) [15].

## Methods

### Study objectives

The goals of the TOEC-Eastern Africa study are:

1. To describe the treatment patterns for ESCC at referral hospitals in Eastern Africa.
2. To investigate the outcomes of different ESCC treatments in Eastern Africa, specifically overall survival, QOL, and safety, with a particular focus on palliative interventions including chemotherapy, radiotherapy with or without chemotherapy, esophageal stenting with SEMS, and supportive care.
3. To examine process measures related to the delivery of ESCC treatment at referral hospitals in Eastern Africa, including time from presentation to initiation of treatment, total time to deliver an intervention, and total number of days hospitalized during and after ESCC treatment.
4. To assess utilization of healthcare resources by ESCC treatment approach, as measured by hospitalization events (total number of times hospitalized and cumulative days hospitalized) and need for subsequent intervention for recurrent or persistent dysphagia.

### Study design

This study is designed as a multi-center, prospective, observational, open cohort study. Initiation of recruitment has been staggered across sites, with the earliest commencing in February 2019 and additional sites beginning recruitment in 2021. Enrollment is scheduled to complete in December 2022, with follow-up to complete in June 2023. In total, approximately 2476 patients are expected to be enrolled in the study. Patients will be followed to collect key clinical and treatment-related data including overall survival and QOL metrics until death or loss to follow up. QOL metrics will be assessed at the time of study enrollment, prior to the initiation of treatment, and during post-treatment follow up (1 month after initiation of treatment, 3 months after initiation of treatment, and then every 3 months until death or loss to follow up). The accrual period is expected to range between 22 and 47 months across sites. Duration of follow up will differ by site due to the staggered initiation of recruitment, however, study participants will be followed for at least 6 months and up to a total of 53 months.

### Study settings

**Fig. 1:**
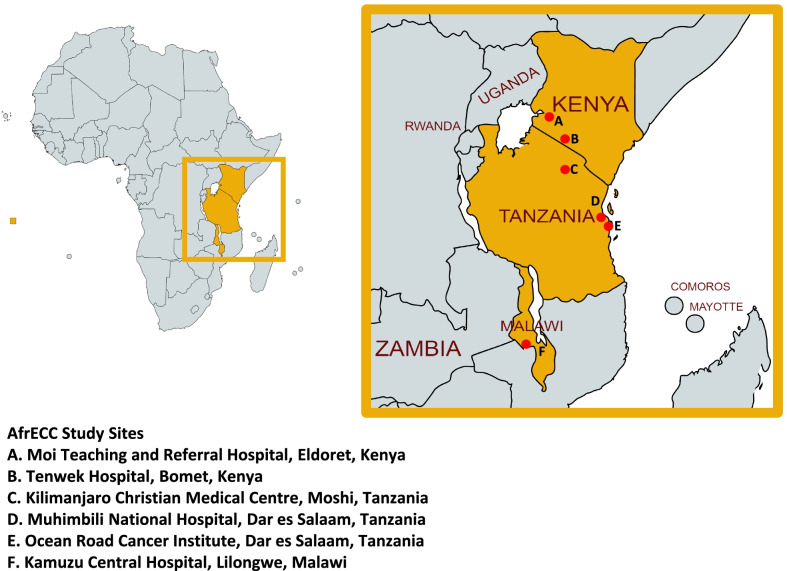
Map of African Esophageal Cancer Consortium (AfrECC) study sites. “Map of African Esophageal Cancer Consortium (AfrECC) sites” published by Van Loon et al. The African Esophageal Cancer Consortium: A Call to Action. J Glob Oncol. 2018 Sep;4:1-9. Licensed under Creative Commons Attribution 4.0. Participant sites modified from original. License: https://creativecommons.org/licenses/by/4.0/

Participating sites are member institutions of AfrECC [15]. A total of six sites across Eastern Africa will participate, including: Ocean Road Cancer Institute (ORCI) and Muhimbili National Hospital (MNH) in Dar es Salaam, Tanzania; Kilimanjaro Christian Medical Center (KCMC) in Moshi, Tanzania; Tenwek Hospital in Bomet, Kenya; Moi Teaching and Referral Hospital (MTRH) in Eldoret, Kenya; and Kamuzu Central Hospital (KCH) in Lilongwe, Malawi (Fig. 1). Each site serves as a referral center for ESCC within the region. A summary of key characteristics and diagnostic and therapeutic services available at each study site is provided in Table 1.

**Table 1:** Characteristics of study sites in Eastern Africa

Site	Tenwek Hospital	Ocean Road Cancer Institute (ORCI)	Muhimbili National Hospital (MNH)	Kamuzu Central Hospital (KCH)	Moi Teaching and Referral Hospital (MTRH)	Kilimanjaro Christian Medical Centre (KCMC)
**Location**	Bomet, Kenya	Dar es Salaam, Tanzania	Dar es Salaam, Tanzania	Lilongwe, Malawi	Eldoret, Kenya	Moshi, Kilimanjaro
**Setting**	Rural	Urban	Urban (two campuses, Upanga and Mloganzila)	Urban	Urban	Urban
**Type of Hospital**	Faith-based hospital	National cancer (specialized) referral hospital	National referral hospital	National referral hospital	National referral hospital	Zonal referral hospital
**Recruitment start date**	February 2019	May 2019	November 2019	June 2021	November 2021	November 2021
**Recruitment end date**^**a**^	December 2022	December 2022	December 2022	December 2022	December 2022	December 2022
**Estimated annual new diagnoses of EC**	250	150	167	300	325	90
**Forecasted annual recruitment**	220	130	150	160	275	80
**Forecasted enrollment (total)**	862	476	472	253	320	93
**Duration of enrollment**	47 months	44 months	38 months	19 months	14 months	14 months
**Diagnostic modalities available**						
Endoscopy	Yes	No	Yes	Yes	Yes	Yes
Pathology	Referred to outside facility	Yes	Yes	Yes	Yes	Yes
X-ray / ultrasound	Yes	Yes	Yes	Yes	Yes	Yes
CT scan	Yes	Yes	Yes	Yes	Yes	Yes
PET scan	No	No	No	No	No	No
EUS	No	No	No	No	No	Mo
**Treatment modalities available**						
Chemotherapy	Yes	Yes	Yes^a^	Yes	Yes	Yes
Radiation						
Brachytherapy (for EC)	No	No	No	No	No	No
EBRT	No	Yes	No	Planned 2022	Yes	Referred to ORCI
Esophageal stents	Yes	No	Yes	Yes	Yes	Yes
Esophagectomy	Yes	No	Yes	No	Yes	No
**Supportive care**						
Availability of TPN	Yes	Yes	Yes	Yes	Yes	Yes
Capacity for gastrostomy tubes	Yes	No	Yes	Yes	Yes	Yes
Intensive care unit	Yes	No	Yes	Yes	Yes	Yes
Palliative care team	Yes	Yes	No	Yes	Yes	Yes

Updated May 2021

*CT* Computed tomography, *EC* Esophageal cancer, *EBRT* External beam radiotherapy, *EUS* Endoscopic ultrasound, *PET* Positron emission tomography, *TPN* Total parental nutrition

^a^Chemotherapy is offered at MNH; most EC patients, however, are referred to ORCI for chemotherapy

In Tanzania, MNH is a public teaching hospital and national referral hospital affiliated with Muhimbili University of Health and Allied Sciences (MUHAS). MNH hosts 1500 beds, admitting 1000 to 1200 inpatients per week and providing care to more than 1000 outpatients per day. Following diagnosis, many of the cancer cases warranting chemotherapy and/or radiation therapy are referred to ORCI, the national cancer referral center in Tanzania, which hosts 273 beds. Muhimbili Academic Medical Center (MAMC) is a recently established public hospital affiliated with MNH. MAMC is located on the Mloganzila campus approximately 30 km from ORCI and MNH. Endoscopy services are available at both MNH and MAMC. KCMC is a public teaching hospital and zonal referral hospital located in Moshi, Tanzania. KCMC hosts 630 inpatient beds and sees an estimated 500-800 outpatients per day. It serves as the teaching hospital for the Kilimanjaro Christian Medical University College and hosts the Kilimanjaro Clinical Research Institute (KCRI), an affiliated research institution.

In Kenya, Tenwek Hospital is a faith-based hospital and tertiary care center located in the southwestern Rift Valley Province in Bomet County. Tenwek Hospital hosts 361 inpatient beds and sees approximately 140,000 annual outpatient visits. MTRH is a 990-bed tertiary referral and teaching hospital in Eldoret, Kenya. The hospital serves residents of the Western Kenya Region, parts of eastern Uganda and South Sudan with a catchment area that covers 22 counties and extends 150 km to the Kenya-Uganda border, and a population of about 24 million people. MTRH has 1200 inpatients at any one time and provides care to 1500 outpatients per day. It has several specialist outpatient clinics under Chandaria Cancer and Chronic Disease Centre (CCCDC). Following diagnosis, oncology patients are referred to Oncology clinic for chemotherapy, radiotherapy or palliative/supportive care. Endoscopy services are available at MTRH and other private hospitals in Eldoret town. MTRH serves as a teaching hospital for Moi University College of Health Sciences and other medical training institutes.

In Malawi, KCH is an 800-bed tertiary referral hospital in Lilongwe. It serves six districts of the central region of Malawi with a population of about 6 million people. The Malawi Ministry of Health plans to open the first Malawi National Cancer Center in Lilongwe in late 2021 on the KCH campus. This will be the first dedicated cancer center offering multidisciplinary, comprehensive cancer care in the country, including radiotherapy, and will serve all of Malawi’s ~ 18 million people. KCH has working relationships, both clinical and research, with the College of Medicine under the University of Malawi and the Malawi College of Health Sciences.

### Outcome assessment

#### Primary endpoint

The primary endpoint for this study will be overall survival (OS), as defined by time elapsed from date of diagnosis to death or last follow up.

#### Secondary endpoints

Secondary endpoints that will be assessed include health-related QOL, as measured by the ‘Modified Roseblatt Index,’ [16] major complications and treatment-related mortality. The ‘Modified Rosenblatt Index’ is a questionnaire assessing patient-reported outcomes previously developed for use among EC patients by Rosenblatt, et al., which has been adapted for use within a resource-limited context (see Table 2) [16]. Parameters evaluated as part of this index are scored on a Likert scale assessing the presence and severity of the following: Dysphagia, Odynophagia, ‘Chest and/or Back pain,’ World Health Organization (WHO) performance status, and ‘Overall Well-being’ since starting treatment. Secondary outcome measures related to dysphagia will also be assessed as dysphagia is commonly a dominant symptom among patients with advanced disease. These secondary measurements include time to achieve any dysphagia relief, time to achieve maximal dysphagia relief, duration of dysphagia relief, and dysphagia-adjusted survival.

**Table 2:** Quality of life metrics as measured by ‘Modified Rosenblatt Index’^a^

	Score				
	0	1	2	3	4
**Dysphagia** Which of the following best describes your ability to swallow?	Normal	Can swallow most foods	Can swallow a soft diet	Can swallow fluids only	Unable to swallow saliva
**Odynophagia** Which of the following best describes the severity of pain you experience when swallowing?	No pain	Mild pain	Moderate pain	Severe pain	–
**Regurgitation** Which of the following best describes how frequently you experience acid reflux, heart burn and/or the sensation of burning in the esophagus?	Never	Infrequent	Frequent	Constant	–
**Chest-back pain** Which of the following best describes your chest and/or back pain?	No pain	Pain relieved by non-narcotics	Pain not relieved by non-narcotics or requiring an opiate medication	–	–
**WHO Performance status** Which of the following best describes your symptoms and activity level on a daily basis?	Fully active, able to carry on all pre-disease performance without restriction	Restricted in physically strenuous activity but ambulatory and able to carry out work of a light or sedentary nature	Ambulatory and capable of all selfcare but unable to carry out any work activities. Up and about more than 50% of waking hours	Capable of only limited selfcare, confined to bed or chair > 50% of waking hours	Completely disabled. Cannot carry on any selfcare. Totally confined to bed or chair
**Overall well-being** Which of the following best describes your quality of life now as compared to before you were treated for esophageal cancer?	“A lot better”	“A little better”	“The same”	“A little worse”	“A lot worse”

^a^Modified quality of life index reprinted with permission from Rosenblatt (2010, Table 1)

#### Exploratory endpoints

Exploratory endpoints related to processes of care will also be measured, including time from presentation to initiation of ESCC treatment, time to deliver intervention (defined as total time elapsed from initiation of treatment to last day of treatment), and total number of days hospitalized during and after an intervention. Measures of healthcare utilization that will be assessed include hospitalization events (total number of times hospitalized and cumulative days hospitalized), and the proportion of patients receiving a subsequent intervention for recurrent or persistent dysphagia. Hospitalization is defined as admittance to the inpatient setting of a hospital (medical or surgical ward or intensive care unit) for a period of ≥24 h per participant report.

### Eligibility criteria

Patients aged ≥18 years with a diagnosis of EC will be eligible for study inclusion. Expanded inclusion criteria will be used as not all patients with suspected EC undergo diagnostic biopsies due to the costs associated with biopsy and pathological review. Diagnostic criteria for EC will include either histopathological confirmation or clinical diagnosis based on barium swallow (esophagram) and/or upper endoscopy without biopsy. This clinical criteria has been shown to have a > 90% pre-test probability for the diagnosis of ESCC in Eastern Africa [17]. Access to a mobile phone is not a requirement for study entry; however, should a study participant lack access, this will be noted on baseline enrollment forms. Patients who have previously received treatment for EC will be eligible for study participation; in these cases, details of prior EC treatment will be abstracted from medical records, if available.

### Recruitment and consent of study participants

Patients who present to participating AfrECC sites with symptoms concerning for or a new diagnosis of EC will be identified by the research team. Since many patients with ESCC are ill and subject to clinical deterioration, we will employ rapid case ascertainment strategies to identify all cases of EC seen for further care. Study teams are embedded in clinical care settings. At designated times each week, research coordinators will use the following strategies to identify eligible patients with suspected diagnoses of ESCC: 1) contact physicians caring for inpatients on both medical and surgical wards and review current ward rosters; 2) review the schedule of endoscopy cases to be performed, including diagnostic and therapeutic procedures (if applicable); 3) review pathology reports (if applicable); 4) contact clinicians seeing patients in the outpatient setting; and 5) review chemotherapy and radiation schedules to identify patients with plans to undergo ESCC treatment (if applicable).

Once potential study participants are identified, they will be approached by a research assistant to assess eligibility and willingness to participate in this study. Study participants will be thoroughly informed about all aspects of the study, including study follow up plans and all regulatory requirements for informed consent. Written informed consent will be obtained prior to study enrollment. The informed consent documents will be available in both English and the local language(s) at each site. Study participants will receive a phone card as a small token of compensation for participation, valued at 5 USD in Kenya and Tanzania and 10 USD in Malawi, in accordance with the local institutional standards.

### Data collection procedures

#### Case report forms (CRFs)

CRFs are comprised of both medical chart abstraction instruments and standardized questionnaires. CRFs collect socio-demographic details (including age, sex, ethnicity, location of permanent residence, highest level of education completed, self-reported annual household income, health insurance status), baseline clinical information, treatment-related data, as well as vital status and QOL metrics over long-term follow up (see Table 3).

**Table 3:** Data collected during TOEC-Eastern Africa study

Collected data	Baseline	Prior to treatment	During treatment	Follow up^**a**^
Sociodemographic				
Age	x			
Sex	x			
Ethnicity	x			
Occupation	x			
Location of permanent residence	x			
Highest level of education completed	x			
Annual household income	x			
Health insurance status	x			
Smoking and alcohol use	x			
Clinical data				
Medical History	x			
HIV status (date of and last CD4 count, use of antiretrovirals)	x			
Date/method of diagnosis	x			
Histopathological data (if applicable)	x			
Endoscopic findings (location/length of tumor)	x			
Imaging (if applicable)^b^	x			
Use of opiate medications for pain control	x	x		x
Hospitalizations			x	x
Vital status (including cause of death, if applicable)			x	x
Treatment				
Prior EC treatment (if applicable)	x			
EC treatment received, including delays/interruptions in treatment and reasons			x	
Complications			x	
Response assessment (if applicable)			x	
Quality of life				
Modified Rosenblatt Index^c^	x	x		x

*EC* Esophageal cancer

^a^Follow up assessments performed 1 month after initiation of treatment, 3 months after initiation of treatment, and then every 3 months until death or loss to follow up

^b^Any imaging modality performed during baseline assessment, including chest x-ray, ultrasound, computed tomography, positron emissions tomography, etc.

^c^The Modified Rosenblatt Index assesses the following: Dysphagia, Odynophagia, ‘Chest and/or Back pain,’ WHO performance status, and ‘Overall Well-being’ since starting treatment

#### Patient interviews

Interviews with study participants will be conducted at the following specified time-points: (i) at time of study enrollment); (ii) prior to the initiation of treatment (within 4 weeks); and (iii) during post-treatment follow up (1 month after initiation of treatment, 3 months after initiation of treatment, and then every 3 months until death or loss to follow up). Research assistants will collect data on sociodemographic characteristics, medical history, and baseline QOL through face-to-face administration of oral questionnaires at the time of study enrollment. QOL assessments are based on the ‘Modified Rosenblatt Index,’ [16] a questionnaire comprised of six patient-reported outcomes assessing for the presence and severity of the following as scored on a Likert scale: Odynophagia, Dysphagia, ‘Chest and/or Back pain,’ WHO performance status, and ‘Overall Well-being’ since starting treatment (see Table 2). Subsequent interviews assessing vital status and QOL will be performed through mobile phone outreach. During post-treatment follow up, research assistants will attempt to contact patients a minimum of three times over the follow up interval before patients are classified as “lost to follow up.” Outreach attempts will be made at varying times of the day on three different days of the week.

#### Chart abstraction

Clinical information will be abstracted from the patient’s medical record at baseline (at time of study enrollment), during treatment(s), and following completion of any treatment(s). Clinical and treatment data collected through chart abstraction is summarized in Table 3. Treatment data will include dates of treatment, procedural details (stenting, esophagectomy, gastrostomy tube placement), radiation treatment regimens, chemotherapy regimens, major treatment- and disease-related complications, best response to treatment (if evaluated), reasons for discontinuing treatment or pursuing supportive care, and date and cause of death. Data will be collected on the time elapsed from diagnosis to initiation of treatment to account for any treatment delays that may explain differences in survival during analyses.

#### Data management

Data will be first collected using paper forms with subsequent entry of de-identified data into Research Electronic Data Capture (REDCap™), a secure web-based application for data storage. All study-related information, including completed paper forms for study participants, will be stored in locked cabinets in a secure location only accessible to authorized study personnel at each study site. Data for each participating site will be stored within a password-protected REDCap™ data access group, with access limited to only authorized personnel from the recruiting AfrECC site. Electronic data capture forms within REDCap™ include data entry fields with programmed checks (‘validation rules’) for range and structure. University of California, San Francisco (UCSF) will serve as the data management coordinating center; however, each site will have access to its own data through this open-source platform.

### Sample size

We aim to recruit all patients with ESCC based on histological confirmation or presumptive clinical diagnosis that meet the eligibility criteria at participating AfrECC sites. We forecast a total accrual of an estimated 2476 study participants across all six sites (see Table 1) over the duration of the study. This estimate is based on annual ESCC cases observed in recent case series at KCH (unpublished data), annual recruitment to a recent case-control study at KCMC [18], as well as annual accrual rates in the present study at ORCI, MNH, and Tenwek since study initiation (Tenwek Hospital, *n* = 220 study participants per year; ORCI, *n* = 130 per year; and MNH, *n* = 150 per year). Of the total study population, we estimate 1102 study participants will be treated with esophageal stenting, 145 with chemotherapy, 464 with radiotherapy, 212 with concurrent chemoradiation, 450 with supportive care alone, and 103 with esophagectomy. Based upon previous data that indicate median OS following stenting is 36 weeks [10], radiotherapy with or without chemotherapy is 27 weeks [13, 19], and chemotherapy is 14 weeks [5, 19], our primary analysis comparing OS of different palliative interventions for ESCC will have the following power to detect differences between each pairwise comparisons based upon alpha of 0.0167 (adjusting for 3 comparisons based on Bonferroni correction): stenting vs. radiotherapy with or without chemotherapy, power = 0.99; stenting vs. chemotherapy, power = 1.00; and radiotherapy with or without chemotherapy vs. chemotherapy, power = 1.00.

### Statistical methods

All patients consenting to study participation with a histopathologically-confirmed or clinical diagnosis of EC will be included in the analyses. Descriptive statistics will be used to describe the proportion of ESCC patients receiving treatment with each modality at participating AfrECC sites. We will assess the associations between demographic/clinicopathologic characteristics and the treatment modalities employed (chemotherapy, radiotherapy, chemoradiation, esophageal stenting with SEMS, esophagectomy, supportive care alone). Categorical variables and continuous variables will be compared among the groups using Pearson’s Chi-squared test, and Analysis of Variance (ANOVA) (or Kruskal Wallis test when normality assumption is not held), respectively. Possible transformation (e.g., logarithm transformation) will be performed if the data is skewed.

OS will be defined in two ways: 1) from the date of initial diagnosis to the date of death or last patient contact; and 2) from date of initiation of treatment to date of death or the date of last patient contact. Kaplan-Meier method will be used to summarize OS and log-rank test will be used to compare survival curves among treatments overall and stratified by the study center. Furthermore, Cox proportional hazards (cph) modeling will be used to account for demographic and clinicopathological factors by including them as covariates. Proportionality of hazards for key covariates will be tested by examining the correlation between time and scaled Schoenfeld residuals and log-log plots of survival. Important covariates that violate the proportionality assumption will be used to stratify the cph model. In many settings in Eastern Africa, long delays can occur between diagnosis and initiation of treatment. To mitigate immortal time bias that can emerge in time-to-event analyses of observational studies, two analytical approaches will be employed. First, in analyses using OS defined by date of diagnosis, the time from diagnosis to the initial treatment will be added as a covariate. Secondly, in analyses of associations between different treatments and OS, treatment status will be included in Cox regression modeling as a time-varying covariate (defined as treated versus untreated). This approach allows for patients to accrue follow up time while awaiting treatment, while also accounting for any events that occur during the ‘untreated’ waiting period [20]. Our primary analysis will examine pairwise comparisons of: stenting vs. radiotherapy with or without chemotherapy, stenting vs. chemotherapy, and radiotherapy with or without chemotherapy vs. chemotherapy. Among patients receiving radiation, outcomes may differ between patients receiving radiotherapy alone as compared to concurrent chemoradiation. Exploratory analyses will examine these subgroups separately.

Cochran-Armitage test will be used to compare symptom metrics assessed using the ‘Modified Rosenblatt Index’ (Dysphagia, Odynophagia, ‘Chest and/or Back pain,’ WHO performance status, and ‘Overall Well-being’ since starting treatment) between each of the different palliative EC treatments (chemotherapy, radiotherapy, chemoradiation, esophageal stenting, basic supportive care) at each time point: 1 month after initiation of treatment, 3 months after the initiation of treatment and then every 3 months thereafter. We will also test for within-subject differences in scores between baseline (or pre-treatment) and each of post-treatment time points by McNemar’s Chi-squared test. In addition, generalized estimating equations (GEE) framework will be applied to analyze this longitudinal QOL data, where the treatment will be considered as the fixed effect and the subject-specific effects are the random effects.

Complications will be summarized as number of patients (%) with 95% confidence intervals and compared between treatment arms by Chi-squared test. Among palliative interventions, secondary outcome measures related to dysphagia will be compared using log-rank test and cph models to adjust covariates, including age, sex and baseline clinical covariates. ‘Time to achieve any dysphagia relief’ will be defined as time from treatment to improvement in dysphagia score of greater than or equal to 1. ‘Duration of dysphagia relief’ will be defined as time elapsed after treatment with any improvement in dysphagia from baseline. ‘Dysphagia-adjusted survival’ will be defined as the total time period with a dysphagia score of 0 to 2.

Measures of healthcare utilization and process measures related to the delivery of ESCC treatment by treatment approach will be examined as exploratory endpoints. Time-to-event measures will be compared among treatment groups by log-rank test. Count of hospitalization events, and total number of days hospitalized during and after ESCC treatment will be compared among treatment groups by ANOVA (or Kruskal Wallis test when normality assumption is not held). Chi-squared test will be used to compare the need for subsequent intervention for recurrent or persistent dysphagia by treatment approach. All analyses will be performed overall and within each site.

All statistical analyses will be performed using Stata or R statistical software. The significance level will be set for 0.05 for all analyses and multiple testing adjustments will be performed by Bonferroni correction.

### Reporting of study results

The study results will be reported in adherence to Strengthening the Reporting of Observational Studies in Epidemiology (STROBE) criteria (see Supplemental Material for checklist) [21].

## Discussions

The TOEC-Eastern Africa study will provide an overview of the therapeutic landscape of ESCC in Eastern Africa. The study will improve our understanding of the outcomes of different treatment strategies for ESCC currently in use throughout the region, including chemotherapy, radiotherapy with and without chemotherapy and esophageal stenting with SEMS. This study will address important an unmet need, as there is currently a paucity of data on the treatment of ESCC in low resource settings, particularly related to QOL [5]. The TOEC-Eastern Africa study will be the first within the region to prospectively compare treatment strategies for ESCC, and the first study in sub-Saharan Africa to evaluate QOL benefits associated with different treatments. Ultimately, findings from this study have potential to inform clinical practice guidelines for the management of ESCC in sub-Saharan Africa and other low resource settings.

Palliative interventions for EC have been an active area of research over the last several decades. Most of this research has taken place in HICs, with several RCTs having investigated best practices for palliation among patients with either ESCC or esophageal adenocarcinoma [9]. These studies highlight the tradeoffs between esophageal stenting with SEMS and the use of radiation-based therapies in patients with advanced disease [22, 23]. SEMS have been shown to provide immediate dysphagia relief, but are prone to failure over time due to stent migration and/or recurrent obstruction. Radiation-based therapies, including external beam therapy (EBRT) with or without chemotherapy and brachytherapy, have delayed effects, but may offer more durable symptom control. Steyerberg et al. developed and validated a prognostic index to help guide treatment selection in this patient population, with the rationale that SEMS may be advantageous for those with short life expectancies [24]. Subsequent efforts to evaluate this index in a South African patient population found that it had poor predictive value in this context, highlighting the challenges of generalizing evidence from clinical trials in HICs to low resource settings [25]. Advanced dysphagia, severe weight loss, and malnutrition are all much more prevalent among patients with EC in these settings. Access to supportive care is often more limited as well. These differences, both in terms of patient characteristics and health system factors, underscore the importance of advancing research on treatment strategies for ESCC in sub-Saharan Africa, with the goal of optimizing treatment delivery in this context. Although RCTs remain the gold standard in comparative effectiveness research, several challenges emerge with this study design in Eastern Africa, including the ethics of randomizing patients who are often in poor general condition, and the time, infrastructure and resources needed for implementing well-designed clinical trials [26, 27].

The TOEC-Eastern Africa study is timely as efforts are currently underway to improve access to oncologic care throughout Eastern Africa as well as other regions in Africa. Historically, treatment approaches for ESCC in Eastern Africa have varied widely, with practice patterns determined by resource availability, as well as patients’ access and financial means. Many cancer referral centers in the region have been able to offer patients with advanced ESCC esophageal stenting with SEMS, radiotherapy with or without chemotherapy, or chemotherapy alone. Few centers host the infrastructure and trained personnel to consider patients for all three treatment modalities. However, access to different treatment modalities is beginning to improve as governments invest in expanding the oncology workforce, developing infrastructure for radiotherapy, and essential medicines for cancer care become more broadly available. Parallel to these efforts, access to SEMS for esophageal stenting has improved in recent years as a result of coordinated efforts within AfrECC to launch the Stent Access Initiative [14]. This program, launched in collaboration with Boston Scientific and the Clinton Health Access Initiative, established the first ever sustainable supply chain of high-quality and affordable SEMS for AfrECC sites in Kenya, Tanzania, Malawi, and Zambia. This access program has been paired with a Training-of-Trainer program focused on building endoscopic skills for SEMS placement without fluoroscopy [28]. As treatment options for ESCC become more widely available for patients across the region, the need to better understand the risks and benefits of the treatments in use and establish evidence-based practice guidelines becomes all that more pressing.

In conclusion, Eastern Africa is impacted by an immense burden of ESCC. Despite being one of the most commonly diagnosed cancers in the region, there is a paucity of evidence to guide treatment decisions in the management of ESCC in this setting. The TOEC-Eastern Africa study leverages existing collaborations within AfrECC to collect robust data on ESCC treatment outcomes at six cancer referral centers in Kenya, Malawi, and Tanzania. Findings from this study have potential to improve our understanding of optimal treatment strategies for ESCC in Eastern Africa and other resource-limited settings. This coordinated prospective cohort study across six sites in three countries is novel and, if successful, may serve as a pragmatic approach to generate context-specific data regarding cancer therapies in sub-Saharan Africa.

### Study status

The study first initiated recruitment at Tenwek Hospital in Kenya in February 2019, followed by Ocean Road Cancer Institute in Tanzania in May 2019. The multi-center study concept was presented by Dr. Geoffrey Buckle at the AfrECC Pre-Conference Meeting at the African Organization for Research and Training in Cancer (AORTIC) International Conference on Cancer in Africa in Maputo, Mozambique (November 5-8, 2019). The study began recruitment at Muhimbili National Hospital in November 2019, Kamuzu Central Hospital in June 2021 and Moi Teaching and Referral Hospital in November 2021. Study recruitment is anticipated to complete December 2022 with follow up completing June 2023. The total duration of the study is estimated to be 53 months.

## Supplementary Information

**Additional file 1. **STROBE Checklist.

## Data Availability

De-identified datasets generated from the current study are available upon reasonable request. Applications to access the datasets should be submitted to the corresponding author. All applications will be reviewed by the corresponding author and the TOEC study site leaders for approval.

## Abbreviations

AfrECC: African Esophageal Cancer Consortium
AORTIC: African Organization for Research and Training in Cancer
CCCDC: Chandaria Cancer and Chronic Disease Centre
CRF: Case reports forms
EBRT: External beam radiotherapy
EC: Esophageal cancer
ESCC: Esophageal squamous cell carcinoma
HDRILBT: High-dose-rate intraluminal brachytherapy
HICs: High-income countries
IRB: Institutional Review Board
IREC: Institutional Research and Ethics Committee
KCH: Kamuzu Central Hospital
KCMC: Kilimanjaro Christian Medical Centre
KCRI: Kilimanjaro Clinical Research Institute
KEMRI: Kenya Medical Research Institute
MAMC: Muhimbili Academic Medical Center
MNH: Muhimbili National Hospital
MTRH: Moi Teaching and Referral Hospital
MUHAS: Muhimbili University of Health and Allied Sciences
NACOSTI: National Commission for Science, Technology and Innovation
NHSRC: National Health Sciences Research Committee
ORCI: Ocean Road Cancer Institute
OS: Overall survival
QOL: Quality of life
RCT: Randomized Controlled Trial
REDCap™: Research Electronic Data Capture
SEMS: Self-expandable metallic stent
TOEC: Treatment Outcomes of Esophageal Cancer
UCSF: University of California, San Francisco
UNC: University of North Carolina
WHO: World Health Organization
